# Arabidopsis *LSH8* Positively Regulates ABA Signaling by Changing the Expression Pattern of ABA-Responsive Proteins

**DOI:** 10.3390/ijms221910314

**Published:** 2021-09-25

**Authors:** Jinpeng Zou, Zhifang Li, Haohao Tang, Li Zhang, Jingdu Li, Yuhong Li, Nan Yao, Yaxing Li, Deguang Yang, Zecheng Zuo

**Affiliations:** 1 College of Agriculture, Northeast Agricultural University, Harbin 150030, China; 17706314607@163.com (J.Z.); 13889216081@163.com (J.L.); 2 Jilin Province Engineering Laboratory of Plant Genetic Improvement, College of Plant Science, Jilin University, Changchun 130062, China; zhang_li18@mails.jlu.edu.cn; 3 Basic Forestry and Proteomics Research Center, Fujian Agriculture and Forestry University, Fuzhou 350002, China; li18834414500@163.com (Z.L.); tanghaohao0987@163.com (H.T.); lyh317@163.com (Y.L.); 18844199632@163.com (N.Y.); fafu_lyx@163.com (Y.L.)

**Keywords:** Arabidopsis, *LSH8*, ABA, seed germination, proteomics, ARPs

## Abstract

Phytohormone ABA regulates the expression of numerous genes to significantly affect seed dormancy, seed germination and early seedling responses to biotic and abiotic stresses. However, the function of many ABA-responsive genes remains largely unknown. In order to improve the ABA-related signaling network, we conducted a large-scale ABA phenotype screening. LSH, an important transcription factor family, extensively participates in seedling development and floral organogenesis in plants, but whether its family genes are involved in the ABA signaling pathway has not been reported. Here we describe a new function of the transcription factor *LSH8* in an ABA signaling pathway. In this study, we found that *LSH8* was localized in the nucleus, and the expression level of *LSH8* was significantly induced by exogenous ABA at the transcription level and protein level. Meanwhile, seed germination and root length measurements revealed that *lsh8* mutant lines were ABA insensitive, whereas *LSH8* overexpression lines showed an ABA-hypersensitive phenotype. With further TMT labeling quantitative proteomic analysis, we found that under ABA treatment, ABA-responsive proteins (ARPs) in the *lsh8* mutant presented different changing patterns with those in wild-type Col4. Additionally, the number of ARPs contained in the *lsh8* mutant was 397, six times the number in wild-type Col4. In addition, qPCR analysis found that under ABA treatment, *LSH8* positively mediated the expression of downstream ABA-related genes of *ABI3*, *ABI5*, *RD29B* and *RAB18*. These results indicate that in Arabidopsis, *LSH8* is a novel ABA regulator that could specifically change the expression pattern of APRs to positively mediate ABA responses.

## 1. Introduction

As a sessile organism, plants need to undergo a complex internal regulation mechanism and environmental signal regulation to survive in adverse and changeable environments [1]. Phytohormone ABA is an important signaling regulator that plays a crucial role in mediating seed germination and maturation, seedling growth, stomatal movement, flowering and stress responses [1,2]. For example, ABA can regulate seed dormancy to prevent premature germination of seeds under stress conditions so that the seeds are able to germinate under suitable conditions, improving the germination rate [3]. These important functions of ABA are derived from the sophisticated regulatory network of ABA [4].

Current research demonstrates that the ABA signaling network in Arabidopsis includes five important components: ABA receptors with PYR1-like (PYL) components, negative regulator type 2C protein phosphatases (PP2C), positive regulator SNF1-related protein kinase 2 (SnRK2), transcription factors of basic leucine zippers (bZIP) and ABA-responsive genes [5]. The signal transduction of ABA in plants occurs in the following pathways. When ABA is deficient, PP2C with phosphatase activity dephosphorylates SnRK2 to inhibit the expression of downstream ABA-responsive genes activated by SnRK2, while in the presence of ABA, the complex of ABA binding to PYR/PYL/RCAR receptors inhibits the phosphatase activity of PP2C, from which SnRK2 is released. The released SnRK2 phosphorylates the downstream transcription factors ABI3/ABI4/ABI5 and ABA-response element binding factors (ABFs), thereby activating the expression of ABA-responsive genes [4,6,7,8,9,10,11]. Numerous previous studies have shown that a large number of transcription factors in the ABA signaling pathway play an indispensable role. For example, ABI3 is a B3-type transcription factor, and ABI5 is a bZIP transcription factor, both of which mediate ABA-induced inhibition of seed germination and initial seedling growth to participate in ABA signal transduction at the seedling stage [12,13,14]. Their loss-of-function mutation leads to the weakening of the inhibitory effect of ABA on seed germination [13,15]. Exogenous ABA significantly induces the expression of ABI5, the overexpression lines of which show a hypersensitivity phenotype to ABA during seed germination and early seedling development [16]. Furthermore, ABI5, as a bZIP transcription factor, can bind to the ABA binding response element (ABRE) in the promoter region of the target genes to activate the target genes’ expression [17]. Additionally, ABF1, ABF2/AREB1, ABF3, ABF4/AREB2 and other transcription factors have been reported to play important roles in the ABA signaling network [18,19], and after the phosphorylation caused by activated SnRK2, ABF/AREB directly binds to the promoter of stress response genes (such as *RD29A* and *RD29B*) to stimulate its transcriptional activity under stress conditions [19,20,21]. These reports suggest that a large number of transcription factors play an important role in the highly complex signaling network of ABA.

*LSH* is a family of transcription factors with diversified functions, the members of which, in turn, are named *LSH1-LSH10*. *LIGHT-DEPENDENT SHORT HYPOCOTYLS 1 (LSH1* was first found in Arabidopsis in 2004 [22], the overexpression of which can enhance the light response of *Arabidopsis thaliana* seedlings and show an obvious short hypocotyl phenotype. All members of the LSH family have a highly conserved Domain of Unknown Function 640 (DUF640) domain, which is also called the Arabidopsis *LSH1* and Oryza G1 (ALOG) domain in the Pfam protein database [22,23]. The DUF640/ALOG domain contains four all-α helices, the additional insertion of a zinc ribbon and a nuclear location signal (NLS) [24]. Proteins with the DUF640/ALOG domain comprise a class of specific transcription factors in plants, with characteristics of binding DNA sequence specificity, transcriptional regulation activity, nuclear localization and homodimer formation, and control plant growth and development in many aspects. Therefore, transcription factor proteins with such a domain often have specific functions [23,25,26,27]. Studies have found that *LSH1* inhibits hypocotyl length in *Arabidopsis thaliana* in a light-dependent manner. The expression of *LSH3* and *LSH4* in the cells of various lateral organs, such as the cotyledon, the leaf and the flower organ, inhibits the differentiation of the boundary organ [25,28,29]. LSH9 interacts with the temperature sensor ELF3 to regulate hypocotyl elongation [30,31]. In addition, proteins of the LSH family can regulate inflorescence structure and flower organ development in other plant species [32]. *LSH* family genes extensively participate in different biological processes in plants, but whether its family genes participate in plant stress response remains unknown.

In this study, we first reported that *LSH8*, a member of the *LSH* family, participates in the ABA signaling pathway as a positive regulator during seed germination and seedling growth and development of Arabidopsis. With further quantitative proteomic analysis, we found that under ABA treatment, *LSH8* specifically regulates the expression patterns of a series of important ABA-related proteins, resulting in an ABA-hypersensitive phenotype of its loss-of-function mutation. The above results reveal the critical role of *LSH8* in the ABA signaling pathway, providing a novel direction for *LSH* family genes to participate in the stress signaling pathway.

## 2. Results

### 2.1. *LSH8* Regulates Seed Germination and the Elongation of Primary and Lateral Root

**Figure 1:**
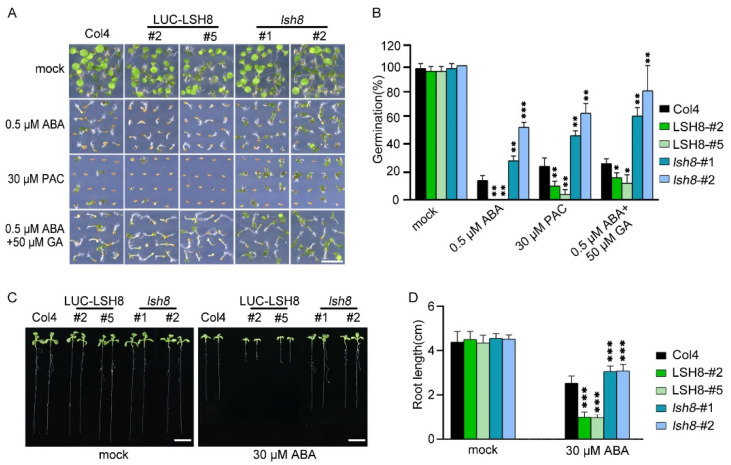
ABA phenotype of *LSH8* overexpression and *lsh8* mutant lines. (**A**) Germination phenotypes of *LSH8* overexpression lines (*LSH8*-#2 and *LSH8*-#5), *lsh8* mutant lines (*lsh8-1* and *lsh8-2*) and wild type (Col4). Seeds were germinated and grown on 1/2 MS (mock) and 1/2 MS containing 0.5 µM ABA, 30 µM PAC, 0.5 µM ABA + 50 µM GA for 5 d, respectively. Scale bar: 1 cm. (**B**) Statistical analysis of germination rate described in (**A**). Data represent mean ± SD of at least 64 seeds. (**C**) Comparison of root length among genotypes on 1/2 MS with or without 30 µM ABA, respectively. Scale bar: 1 cm. (**D**) Statistical analysis of the differences in root length among the genotypes shown in (**C**). Data are shown as mean ± SD (*n* > 10). Asterisks in (**B**,**D**) indicate statistically significant differences compared with wild-type Col4: *, *p* < 0.05; **, *p* < 0.01; ***, *p* < 0.001 (Student’s *t*-test).

*LSH* family genes are reported to be expressed in hypocotyl and flower organs. They are important for the growth and development of plants, but their function in the hormone signaling network remains unknown. To understand the function of *LSH8* in the ABA signaling pathway, we obtained 35S::LUC-*LSH8* overexpression lines *LSH8*-#2 and *LSH8*-#5, *lsh8* mutant lines *lsh8-1* (SALK_024841) and *lsh8-2* (CS845710). Gene expression analysis confirmed that the expression of *LSH8* significantly decreased in *lsh8* mutant lines and markedly increased in *LSH8* overexpression lines, compared with wild-type Col4 (Figure S1). Through phenotypic analysis, we found that without ABA treatment, the seed germination of different genotypes was almost identical, while with 0.5 µM ABA treatment, the seed germination of Col4 was inhibited, and that of *LSH8* overexpression lines *LSH8*-#2 and *LSH8*-#5 was strongly inhibited. However, ABA inhibition on the seed germination of *lsh8* mutant lines *lsh8-1* and *lsh8-2* were obviously attenuated. Thus, *LSH8* overexpression lines were recognized as ABA sensitive and *lsh8* mutant lines insensitive (Figure 1A). As the seed germination is under the joint regulation of the hormones ABA and GA, during which GA promotes seed germination, presenting the opposite effect of ABA, we used GA biosynthesis inhibitor paclobutrazol (PAC) to verify the *LSH8* phenotype and found that under the PAC treatment condition, the seed germination of different genotypes was identical to that under ABA treatment. At the same time, we applied an appropriate amount of GA on ABA treatment conditions, the result of which showed that GA could weaken the inhibitory effect of ABA on seed germination (Figure 1A). To summarize, the above results indicate that ABA and GA simultaneously participate in the process of seed germination. More importantly, we identified a new positive regulator *LSH8* in the ABA signaling pathway. Further statistical results of germination rate showed that under either ABA or PAC treatment, the germination rate of *lsh8* mutant lines was significantly higher than that of Col4, while the germination rate of *LSH8* overexpression lines was significantly lower. Additionally, under ABA treatment with a moderate amount of GA, the seed germination rate of *LSH8* overexpression lines and *lsh8* mutant lines was improved (Figure 1B).

Since the *LSH8*-mediated ABA signaling pathway participates in the regulation of seed germination, whether *LSH8* is involved in ABA-mediated seedling root elongation is another issue of concern. After growing on 1/2 MS medium without ABA for 4 days, the seeds of different genotypes were transferred to 1/2 MS medium with ABA for another 4 days. The root length phenotype and root length measurements showed that when compared with wild-type Col4, the primary roots of the overexpression lines *LSH8*-#2 and *LSH8*-#5 were significantly shorter, but the primary roots of mutant lines *lsh8-1* and *lsh8-2* were significantly longer, and their lateral roots were obviously increasing (Figure 1C,D), showing that *LSH8* promotes ABA’s effect on inhibiting the primary root elongation and lateral root development.

### 2.2. The Prediction of Upstream Element of LSH8 and the Expression of LSH8 Regulated by ABA

In the ABA signaling pathway, ABREs can be recognized by specific transcription factors to activate the expression of ABA downstream related response genes. Most of the promoter region of ABA response genes contains conserved G-box-like *cis*-elements, ABREs (PyACGTGG/TC). Previous studies have shown that genes successfully activated and expressed by ABA require multiple ABREs or one ABRE bound to several coupling elements (CEs). Analyzing the data of PlantCare, we found that the promoter region of *LSH8* contained two ABREs and one G-box element (Figure S2).

**Figure 2:**
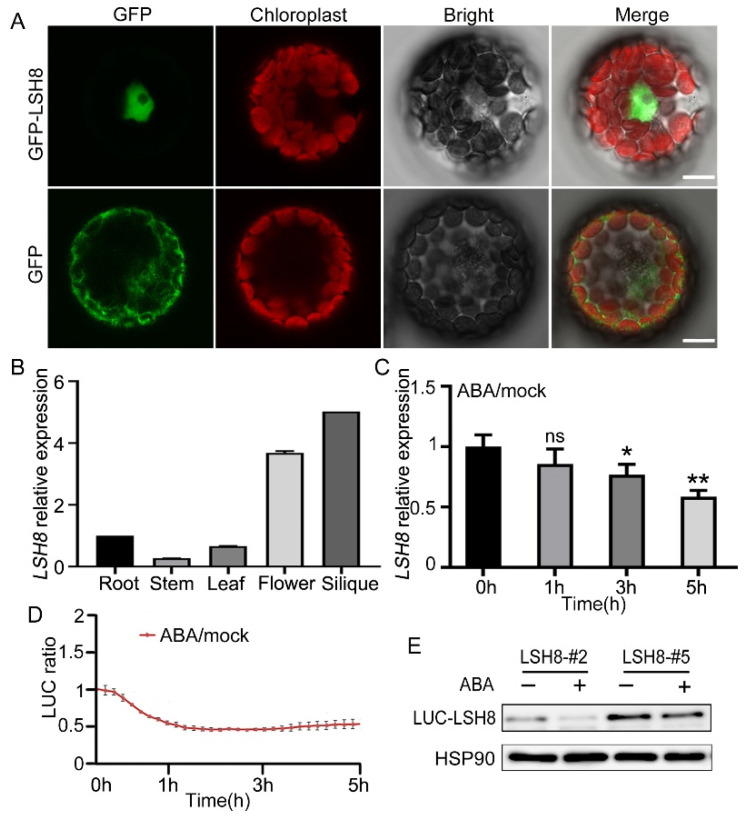
Expression pattern analysis of *LSH8*. (**A**) Subcellular localization analysis of GFP and GFP-*LSH8* in Arabidopsis wild-type Col4 protoplasts. The channels from left to right are GFP, Chloroplast, Bright and Merged channels, respectively. Scale bar: 10 µm. (**B**) Tissue-specific expression of *LSH8*. Various tissues of wild-type Col4 were grown under normal conditions, and the *LSH8* expression was determined by qPCR. Data are shown as mean ± SD (*n* = 3). (**C**) qPCR analysis of *LSH8* transcriptional level under ABA treatment. Five-day-old Col4 seedlings were treated with 100 µM ABA for 0–5 h. Data are shown as mean ± SD (*n* = 3). (**D**) LUC signals in 5-d-old LUC-*LSH8* overexpressing seedlings treated with 100 µM ABA. Signals were detected every 10 min, and the detecting period is 5 h. Data are shown as mean ± SD (*n* = 3). (**E**) Immunoblot analyzing the ABA-induced decline of *LSH8* protein in the LUC-*LSH8* overexpressing lines. Whole seedlings of 5-day-old LUC-*LSH8* overexpression lines were treated with 100 µM ABA for 5 h. The expression of LUC-*LSH8* fusion protein was detected by immunoblotting with an anti-LUC antibody. HSP90 was used as loading control. Asterisks in (**C**) indicate statistically significant differences compared with normal conditions (0 h): ns, *p* > 0.05; *, *p* < 0.05; **, *p* < 0.01 (Student’s *t*-test).

With ePlant (http://bar.utoronto.ca/eplant (accessed on 25 August 2020)), we predicted that *LSH8* was localized in the nucleus. Further protoplast subcellular localization experiment showed that *LSH8* was localized in the nucleus (Figure 2A). Additionally, to analyze the *LSH8* expression in different tissues, we detected the root, the stem, the leaf, the flower and the silique of Arabidopsis by qPCR, the result of which showed that the highest expression of *LSH8* happened in the flower and the silique and the lowest in the stem (Figure 2B). Previous studies have found that *PhLSH7b* of petunia, a homologous gene of *LSH8* in Arabidopsis, regulates plant flowering, but the function of *LSH8* in Arabidopsis is still unknown. To study the function of *LSH8*, we checked the public microarray data (http://bar.utoronto.ca/efp/cgi-bin/efpWeb.cgi (accessed on 13 September 2020)) and found that the expression of *LSH8* may be affected by ABA during seed germination. To confirm this expression pattern, we detected the changes in the transcriptional level and protein level of *LSH8* in wild-type Col4 under ABA treatment. The results showed that the transcriptional level of *LSH8* started to have a gradual decrease after 1.5 h of ABA treatment (Figure 2C). With additional Luciferase assay and Western blot detection, we found that the protein level of *LSH8* from *LSH8* overexpression lines also showed a downtrend under ABA treatment (Figure 2D,E). The above results indicated that ABA inhibited the expression of *LSH8* at both transcriptional and protein levels.

### 2.3. Quantitative Proteomic Analysis

**Figure 3:**
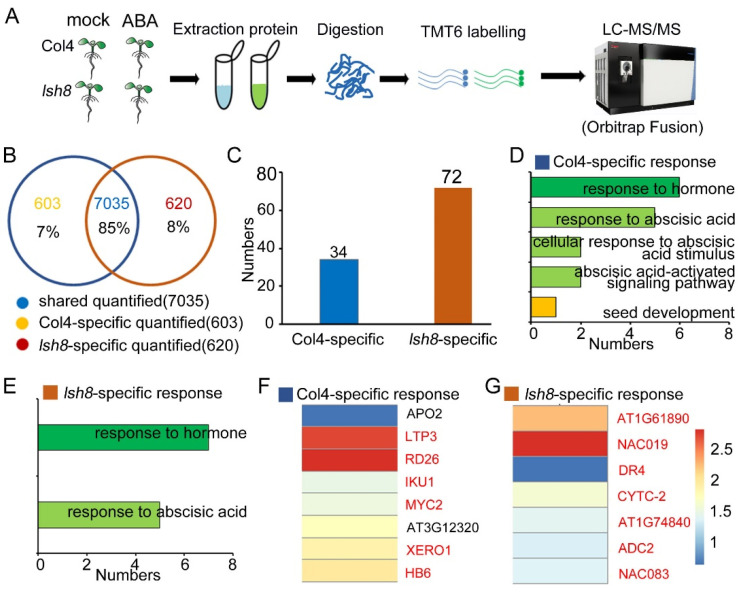
Quantitative proteome analysis of regulatory protein expression profiles by *LSH8* in response to ABA. (**A**) Experimental design of quantitative proteome analysis. Five-day-old Col4 and *lsh8* mutant seedlings were treated with or without 100 µM ABA for 5 h. The protein was extracted in three biological replicates for each sample group. (**B**) Venn diagram showing the number of specific and shared quantified proteins between Col4 and *lsh8* mutant. (**C**) The number of ARPs in Col4 specific quantified proteins and *lsh8* mutant-specific quantified proteins in (**B**). (**D**,**E**) Gene Ontology (GO) enrichment analysis of the Col4 specific ARPs and *lsh8* mutant-specific ARPs shown in (**C**). (**F**,**G**) The heatmap showing the expression patterns of ABA-related proteins that are specifically quantified and responsive to ABA in Col4 or *lsh8* mutant. The red color proteins are ARPs previously reported.

In the above experiments, we found that *LSH8* overexpression lines showed an ABA-hypersensitive phenotype, and *lsh8* mutant lines showed an ABA-insensitive phenotype. For a further study of *LSH8* function in the ABA signaling network, we conducted TMT-based proteomic analysis (Figure 3A, Tables S2 and S3). By comparing with the Arabidopsis thaliana.TAIR 10.31.pep.all database, we found that 76,295 peptides were identified in Col4, including 63,166 specific peptides, corresponding to 9161 proteins (Figure S3A); 75,452 peptides were identified in the *lsh8* mutant, including 62,555 specific peptides, corresponding to 9116 proteins (Figure S3D). More than 79% of the 9161 proteins identified in Col4 contained more than two unique peptides, and more than 80% of the 9116 proteins identified in the *lsh8* mutant contained more than two unique peptides (Figure S3B,E). The protein coverage rates of the proteins identified in Col4 and the *lsh8* mutant with the size of 1–10 kDa, 10–20 kDa, 20–30 kDa, 30–40 kDa, 40–50 kDa, 50–60 kDa, 60–70 kDa, 70–80 kDa, 80–90 kDa, 90–100 kDa and > 100 kDa were 1%, 8%, 14%, 16%, 15%, 14%, 9%, 6%, 5%, 3% and 10%, respectively, and the most identified proteins were in the range of 20–80 kDa (Figure S3C,F). Additionally, we found that the Pearson correlation coefficient of the three biological replicates in the proteome experiment was more than 0.99, indicating the repeatability of our experiment (Figure S4A–D). Principal component analysis (PCA) showed that the contribution ratios of PC1 and PC2 were 58.22% and 1.47%, respectively. Samples of different materials were distributed differentially on PC1 and PC2 under different treatment conditions, certifying the diversity of the experimental materials and the variability of experimental treatment conditions (Figure S4E). We next defined the proteins with the expression fold-change ratio >1.3 or <0.77 and *p* < 0.05 as ABA-responsive proteins (ARPs). According to these two criteria of fold-change ratio and *p*-value, we identified 263 ARPs in Col4, including 60 upregulated and 203 downregulated, and in the *lsh8* mutant, we identified 636 ARPs, of which 428 were upregulated and 208 downregulated (Figure S4F). To classify and describe these ARPs in detail, we used a scatterplot and heatmap to present the specific distribution of ARPs (Figure S5).

### 2.4. Functional Analysis of ARPs

We obtained the expression patterns of 8258 proteins with ABA treatment, which fell into two classes: Class I was of specific quantified proteins in Col4 and *the lsh8* mutant, and Class II was of Col4, and the *lsh8* mutant shared quantified proteins with different ABA expression patterns (Tables S4 and S5).

In Class I, 603 proteins (7%) were specifically quantified in Col4, 620 proteins (8%) were specifically quantified in the *lsh8* mutant, and 7035 proteins (85%) were shared quantified in Col4 and the *lsh8* mutant (Figure 3B). Among the specifically quantified proteins, 34 ARPs were specifically quantified in Col4, just half the number of ARPs specifically quantified in the *lsh8* mutant, that is 72 ARPs (Figure 3C). We then figured that the ARPs especially quantified in Col4 and the *lsh8* mutant were ARPs specifically regulated by *LSH8*, being involved in ABA-responsive pathway and seed growth and development pathway (Figure 3D,E), and among which the expression patterns of some important ABA-related proteins were identified to change (Figure 3F,G).

**Figure 4:**
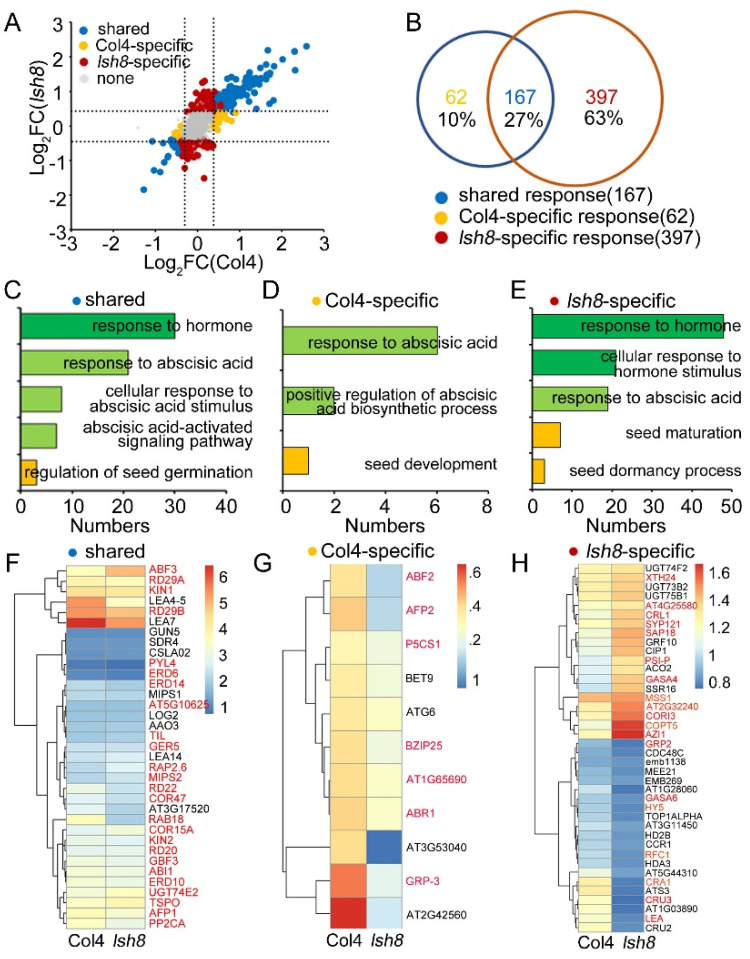
*LSH8*′s regulation of proteome changes in response to ABA. (**A**) Scatterplot showing plots of Log_2_(FC) of wild-type Col4 (*x*-axis) versus Log_2_(FC) of *lsh8* mutant (*y*-axis). The dash lines indicate Log_2_(FC) = ±0.38. (**B**) Venn diagram depicting shared ARPs, Col4-specific ARPs and *lsh8* mutant-specific ARPs determined by proteomics. (**C**–**E**) Gene Ontology (GO) enrichment analysis of the shared ARPs, Col4-specific ARPs and *lsh8* mutant-specific ARPs shown in (**B**). (**F**–**H**) The heatmap showing the ABA-related proteins expression patterns of shared ARPs, Col4-specific ARPs and *lsh8* mutant-specific ARPs shown in (**B**). The red color proteins are ARPs previously reported.

In Class II, we divided 7035 proteins shared quantified in Col4 and the *lsh8* mutant into four groups: ARPs shared regulated by Col4 and the *lsh8* mutant, ARPs specifically regulated by Col4, ARPs specifically regulated by *lsh8* mutant, and proteins neither regulated by both (Figure 4A). There were altogether 626 ARPs regulated by Col4 and the *lsh8* mutant. Among them, 167 ARPs (27%) were jointly regulated by Col4 and the *lsh8* mutant, responding to ABA treatment but not specifically regulated by *LSH8*. With GO enrichment analysis, we identified that these ARPs participated in the ABA response pathway and regulated seed germination pathway (Figure 4B,C). Additionally, 62 (10%) ARPs among the total 626 ARPs were specifically regulated by Col4, and 397 (63%) ARPs were specifically regulated by the *lsh8* mutant, six times higher than those regulated by Col4 (Figure 4B). The ARPs specifically regulated by Col4 and the *lsh8* mutant, respectively, were ARPs specifically regulated by *LSH8* under ABA treatment, which were found to be involved in the ABA response pathway, seed growth and germination pathway and seed dormancy pathway (Figure 4D,E). Simultaneously, the expression pattern of some important ABA-related proteins was specifically regulated by *LSH8* was found to change (Figure 4F–H).

**Figure 5:**
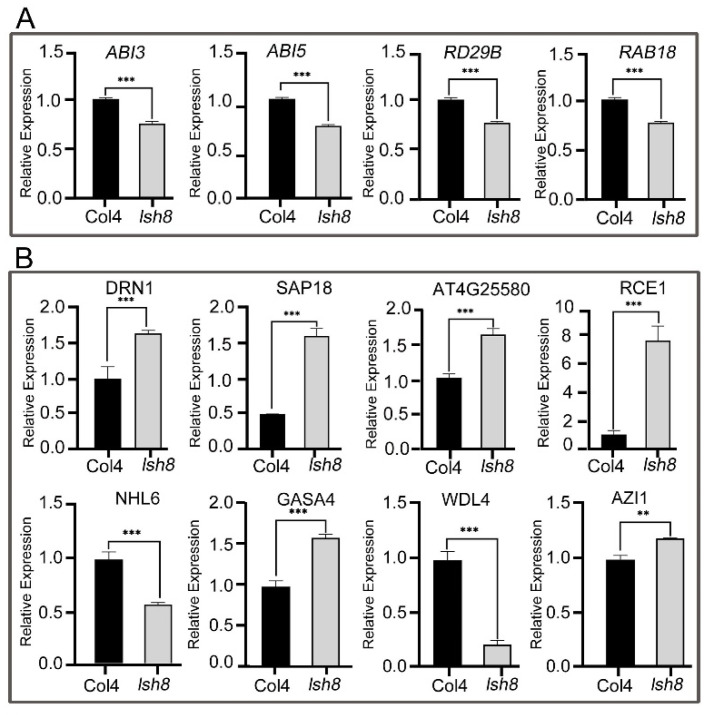
Expression of ABA-related genes and *LSH8* specifically regulated ARPs in Col4 and *lsh8* mutant seedlings under exogenous ABA treatment. The 5-day-old seedlings of Col4 and *lsh8* mutant were transferred to 1/2 MS medium with 100 µM ABA for 5 h, and then the seedlings were harvested immediately for qPCR. (**A**) The expression levels of ABA-related genes. (**B**) The expression levels of *LSH8* specifically regulated ARPs. Data are shown as mean ± SD (*n* = 3). The asterisks in the picture indicate statistically significant differences compared with relevant wild-type Col4: **, *p* < 0.01; ***, *p* < 0.001 (Student’s *t*-test).

In addition, the results of our experiment showed that the ABA downstream response proteins ABI3, ABI5, RD29B and RAB18 exist in ARPs jointly regulated by Col4 and the *lsh8* mutant, with different expression patterns. With a further qPCR analysis. We found that under ABA treatment, the expression of *ABI3*, *ABI5*, *RD29B* and *RAB18* in the *lsh8* mutant was lower than that in Col4 (Figure 5A), indicating that the expression of ABA downstream response genes will be affected in the loss-of-function mutation of *LSH8*. We then detected the expression of the 11 ARPs selected from the proteome at the transcriptional level, and the expression trend of 8 genes was consistent with the change of protein expression in the proteome (Figure 5B). Thus, we speculated that the variation of the expression pattern of these ABA-responsive genes results in the insensitive phenotype of the *lsh8* mutant to ABA.

### 2.5. Interaction Network of *LSH8*-Specific ARPs

**Figure 6:**
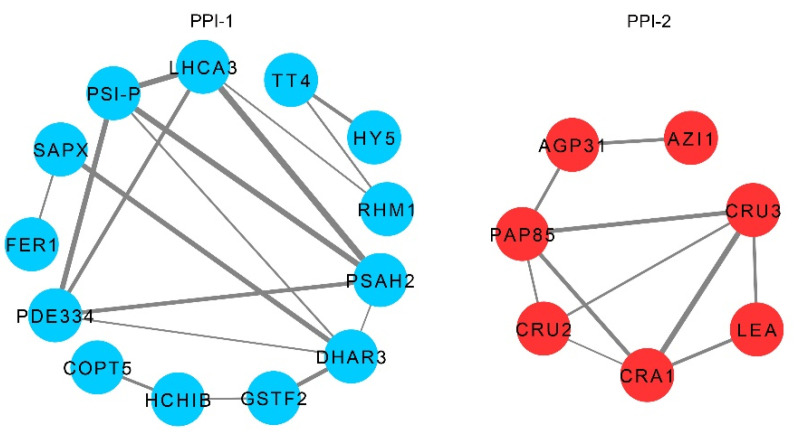
PPI network analysis by STRING. These proteins in the module were ARPs specifically regulated by *LSH8*. Two PPI networks were obtained. Circular nodes represent ARPs, and edges represent protein–protein (ARPs) associations.

In order to clarify the interaction among ARP-regulated pathways in the *lsh8* mutant, STRING analysis was used to generate an interaction network among the ABA-related proteins of the ARPs. With serious consideration, we selected 43 ARPs involved in the ABA signaling pathway in the *lsh8* mutant for a protein–protein interaction (PPI) network analysis, including 30 upregulated and 13 downregulated, all of which were specifically regulated by *LSH8*. The PPI network analysis showed two independent PPIs (Figure 6). Proteins of HCA3, PDE334, PSI-P, PSAH2 and DHAR in the first PPI lay in the position of high connectivity and were regulated by ABA in the *lsh8* mutant. Proteins of PAP85, CRU3, CRU2, CRA1 and LEA occupied the center of the second PPI, closely related to each other. Their expression was all reduced. All these results are consistent with previous results that genes such as *LEA* (AT2G21490), *CRU2* and *CRU3*, *CRA1* and *PAP85* play an important role in maintaining seed dormancy.

## 3. Discussion

Phytohormone ABA is essential in regulating seed germination and early seedling growth and development [33]. The ABA regulatory network of plants is very complicated, and it mainly undergoes regulation on the transcriptional level and protein level [34]. Therefore, the identification of new regulatory factors in ABA signaling significantly improves this regulatory network. In this study, we found a new positive regulatory factor, *LSH8*, in the ABA signaling pathway.

In analyzing the phenotype of *LSH8*, we performed the ABA treatment with different concentrations and found a consistent result with previous research [35,36], showing that the ABA phenotype in *LSH8* overexpression lines and *lsh8* mutant lines is obvious under the treatment of 0.5 μM ABA. Previous studies have shown that ABA inhibits germination, while GA promotes germination [37,38,39]. To further verify the function of *LSH8*, we selected PAC, a GA synthesis inhibitor with the same effect as ABA, and conducted different PAC treatment concentrations simultaneously. We found that the PAC phenotype in *LSH8* overexpression lines and *lsh8* mutant lines was obvious under the treatment of 30 μM PAC, showing the same phenotype as that in ABA treatment. After the ABA treatment, we added an appropriate GA treatment and found that it weakened the inhibition of ABA on seed germination, regardless of phenotype or germination rate statistics (Figure 1A). This result indicates that phytohormone ABA and GA have an antagonistic effect on the function of seed germination. Our experiments verified the function of the positive regulatory factor *LSH8* in the ABA signaling pathway. Furthermore, we speculated that *LSH8* may also participate in the related functions of the GA signaling pathway, providing a new direction for future research on other functions of *LSH8*.

*LSH8* is a member belonging to the family of *LSH* transcription factors in plants [22,24]. In this study, we found that ABRE elements exist in the promoter region of *LSH8* (Figure S3A). ABRE element is the main *cis*-regulatory element of ABA-dependent gene expression, so it is speculated that *LSH8* is involved in the ABA signaling pathway. In our study, the transcriptional level of *LSH8* was regulated by ABA (Figure 2C). Furthermore, the protein level of *LSH8* was also regulated by ABA (Figure 2D,E), and both transcriptional and protein levels decreased. However, *lsh8* mutants showed an insensitive phenotype to ABA in seed germination, primary root and lateral root development (Figure 1A,C), revealing that *LSH8* plays a positive regulatory role in ABA signaling. Therefore, under ABA treatment, the transcriptional and protein level of *LSH8* may undergo negative feedback regulation. Previous studies have shown that ABRE-binding factors (ABFs) can combine with the promoter of *PP2C* to stimulate the expression of *PP2C* to negatively feedback regulate ABA signaling [40,41]. These studies have also found that *ABI5* positively regulates the expression of *MOTHER OF FT AND TFL1 (MFT)*, while *MFT* simultaneously inhibits the expression of *ABI5* to negatively feedback regulate ABA signaling [42]. Thus, we speculated that a similar negative feedback regulation mechanism may also be found in *LSH8* in the ABA signaling pathway, leading to the low expression level of *LSH8* under ABA treatment. This provides a direction for the study of how *LSH8* accurately acts on the ABA signaling pathway in the future.

Through quantitative proteomic analysis, we found that the *lsh8* mutant and Col4 had different protein response patterns responding to ABA (Figure 3A and Figure 4A), which may be the key to *LSH8*’s involvement in the ABA signaling pathway. The shared ARPs, such as RD29A, RD29B, RD22 and RAB18, existed in Col4 and the *lsh8* mutant. As downstream proteins in the ABA signaling pathway, these ARPs were often used as important marker proteins in plants responding to ABA [43,44,45]. We found that the content of these ARPs increased under ABA treatment, but the increased number of these ARPs in Col4 was significantly higher than that in the *lsh8* mutant, indicating that some ARPs can still respond to ABA in the *lsh8* mutant, albeit with the degree reduced. This result shows that *LSH8* weakens the function of ABA-related proteins, rather than completely cutting off their functions.

We found 62 specific ARPs (Figure 4B) in Col4, among which 11 were previously reported proteins involved in the ABA signaling pathway, such as AFP2, AT3G53040 and GRP-3. The expression of AFP2, a negative regulator in the ABA pathway, increased under ABA induction [46] AT3G53040, a putative LEA protein, was induced by ABA to hinder seed germination and promote seed dormancy [47]. GRP-3 was also induced by ABA to regulate root length [48]. Consistent with previous reports, the protein levels of AFP2, AT3G53040 and GRP-3 were also found to be increased with the inducement of ABA in Col4, but the expression of these proteins in the *lsh8* mutant was no longer induced by ABA—that is, proteins regulated by ABA under normal conditions are no longer affected by ABA due to *LSH8* deficiency, which was speculated as one of the reasons for the decrease in the *lsh8* mutants’ sensitivity to ABA. Furthermore, the change pattern of the other 52 unreported ARPs was also speculated as one of the reasons for the decrease in the *lsh8* mutant’s sensitivity to ABA.

Some specific ARPs in the *lsh8* mutant can be divided into two groups. The first contains seven ARPs specifically quantified by the *lsh8* mutant, including AT1G61890, NAC019, DR4, CYTC, AT1G74840, NAC083 and ADC2 (Figure 3F). The other is constituted by the shared proteins quantified by Col4 and the *lsh8* mutant, which are APRs only presenting their specificity in the *lsh8* mutant (Figure 4H), indicating that *LSH8* could inhibit the response of these proteins under ABA treatment. With GO enrichment analysis, we found that some proteins were directly involved in regulating the ABA pathway and the seed growth and development pathway, such as AZI1, which regulates the root length of Arabidopsis [49,50], responding to ABA after *LSH8* deficiency. Other proteins involved in seed storage, such as LEA family proteins AT5G44310 and LEA (AT2G21490) [51], CRU2 and CRU3 [52,53,54,55,56], ATS3 [51,57], AT1G03890 [52] and CRA1 [54,55], could also respond to ABA following *LSH8* deficiency. Therefore, it was speculated that *LSH8* may also play an important role in the ABA maintenance of seed dormancy.

## 4. Conclusions

LSH, an important transcription factor family, plays a role in seedling development and floral organogenesis in plants. This study, for the first time, concludes that the LSH transcription factor family protein *LSH8* participates in the ABA signaling pathway in Arabidopsis. *LSH8* plays a positive regulatory role in ABA signaling, but the transcriptional level and protein level of *LSH8* were downregulated by ABA. This finding indicates that *LSH8* experiences negative feedback regulation after exogenous ABA treatment. Furthermore, *LSH8* induces the change of protein response patterns under ABA treatment. *LSH8* upregulates some shared ARPs existing in Col4 and the *lsh8* mutant, such as RD29A, RD29B, RD22 and RAB18, which are marker proteins in the ABA signaling pathway. Moreover, *LSH8* affects some proteins from specific ARPs in Col4 or the *lsh8* mutant, and these proteins are involved in the ABA pathway, the seed growth and development pathway and seed dormancy. Therefore, *LSH8* is an important factor in the ABA signaling pathway.

## 5. Materials and Methods

### 5.1. Plant Materials and Growth Condition

The *A. thaliana* accession, Col4, was used as the wild type in this study. To generate 35S::LUC-*LSH8* overexpression plants, the full-length cDNAs of *LSH8* behind the LUC tag sequences were cloned into the binary vector pEGAD-LUC by In-Fusion Cloning methods [58,59]. The construct was used for transformation (via Agrobacterium strain Agl0) by the floral-dip method [60]. Transformed plants were screened by growth on Basta-containing medium, and homozygous T3 transgenic lines were used for further analyses. The T-DNA insertion lines for *LSH8* (AT1G16910) were obtained from the Arabidopsis Biological Resource Center (https://abrc.osu.edu/ (accessed on 22 May 2020)) with the following seed stock numbers: SALK_024841 and CS845710. PCR-based screening was used to identify homozygous lines for T-DNA insertions in *LSH8*. The *LSH8*-specific primers, designed by the SIGNAL T-DNA verification primer design program, were used in combination with T-DNA left border primers. The primers used for genotyping *LSH8* overexpressing lines and the *lsh8* mutant are listed in Table S1.

Plants were grown in normal conditions at 22 °C under 60% relative humidity with a photoperiod of 16 h/8 h (light/dark) and 120 µmol m^−2^s^−1^. All seed lots used for experimental material were harvested concurrently [61].

### 5.2. Seed Germination Assays

The seeds of different genotypes used for the germination test were harvested in the same conditions of plants grown at 22 °C under long days (16 h/8 h). Seeds were harvested and stored in dry conditions for at least 5 weeks before the germination test. The germination of the seeds from wild-type Col4, *lsh8* mutants (*lsh8*-1/*lsh8*-2) and *LSH8* overexpression lines (*LSH8*-#2/*LSH8*-#5) were determined as described previously [36]. Briefly, seeds were sterilized with 10% NaClO (Sigma-Aldrich, Saint Louis, USA) for 5 min and washed five times with ddH_2_O. Subsequently, the sterilized seeds were sown on 1/2 MS medium (pH = 5.7) containing 0.7% (*w/v*) Agar contained with 0.01% DMSO (as Mock), 0.5 μM ABA (Sigma-Aldrich), 30 μM PAC (Sigma-Aldrich) and 50 μM GA3 (Sigma-Aldrich) [35,61,62,63]. All the seeds were kept at 4 °C/dark for 3 days for stratification and transferred to 16 h/8 h light/dark conditions at 22 °C for germination. The germination event was defined as the first sign of radicle emergence, and germination rate was recorded daily for five sequential days [37]. At least 64 seeds for each line were used in three biological replicates.

### 5.3. Root Length Analysis

For the root length assay, the seeds from each genotype were germinated vertically on the 1/2 MS (0.7% Agar) for 3 days. Then, approximately 20 seedlings of each genotype showing similar root lengths were transferred to a 1/2 MS medium contained with or without 30 µM ABA for 7 days. The root length of each line was determined after the transfer to 1/2 MS medium for 7 days [61,64,65,66]. Three biological replicates were used.

### 5.4. Confocal Microscopy

The full-length CDS of *LSH8* was amplified using *LSH8*-specific primers (Table S1) and then inserted into the pCAMBIA3301-GFP vector to generate a transient expression vector of the GFP-*LSH8* fusion protein. The plasmids of GFP-*LSH8* and GFP were transferred into Arabidopsis wild-type Col4 protoplasts, then the protoplasts were cultured for 18 h and photographed through a Leica/TCS SP8 confocal microscope (Leica Microsystems, Wetzlar, Germany) with the following conditions: GFP, 488 nm, 63× oil objective [67].

### 5.5. Luminescence Measurement

LUC-*LSH8* overexpression lines were sowed in a 96-well plate containing 1/2 MS medium with 0.25% (w) sugar and 0.4% (w) Agar, with 10 seeds per well for each individual line. Seedlings were transferred to darkness for LUC activity detection by adding 1 mM D-luciferin with or without 100 µM ABA. LUC signals were detected every 10 min, with a detecting period of 5 h.

### 5.6. Quantitative Real-Time PCR (qPCR) Analysis

Total RNA of different genotypes was isolated with the RNeasy Plant Mini Kit (QIAGEN, Dusseldorf, Germany) from 7-day-old seedlings. Then, 1 μg of total RNA was used for reverse transcription reaction. All qPCR experiments were performed on the real-time PCR system (Applied Biosystems 7500, Waltham, MA, USA) using the TB GREEN Premix Ex Taq kit (Takara, Kyoto, Japan). *ACT2* was used as the internal control to analyze the relative expression levels of genes, and parameters in qPCR were performed in triplicate experiments. Relative expression levels of ABA-related genes were calculated using the 2^−ΔΔ*C*t^ method [68,69].

### 5.7. Protein Extraction, Specific-Antibody Preparation and Western Blotting

For every 0.1 g of fresh weight germinating seedlings, each line sample was mixed with 200 μL of protein extraction buffer (50 mM Tris-HCl, pH = 7.5, 150 mM NaCl, 2.5 mM EDTA, PH 8.0, 1 mM DTT, 1% Nonidet P-40). Then, 100 μL of 4× sample buffer was added to each line, and the sample was vortexed immediately. Samples were then boiled at 95 °C for 5–10 min and centrifuged for 10 min. The supernatant was transferred to a new 1.5 mL tube, from which samples were loaded onto SDS-PAGE for immunoblotting. Anti-LUC (1:3000 dilution, Abclonal, Wuhan, China) was used as the first antibody, and an HRP-conjugated anti-rabbit IG (H + L) (1:8000 dilution, MBL, Beijing, China) was used as the second antibody. HSP90 antibody (Beijing Protein Innovation, AbM51099-31-PU, Beijing, China) was used as the loading control.

### 5.8. Protein Sample Preparation

Approximately 0.5 g of each mixed sample was extracted from ground material. The trichloroacetic acid (TCA)/acetone method was used for total protein extraction, and the filter-aided sample preparation (FASP) method was used for total protein digested [70,71]. Then, the trypsin was added at a 1:50 trypsin-to-protein mass ratio for protein digestion overnight at 37 °C for 12 h. After trypsin digestion, peptides were reconstituted in 1 M triethylammonium bicarbonate (TEAB) and were labeled using TMT6-plex kits according to the manufacturer’s protocol (Thermo Fisher Scientific, Torrance, CA, USA).

### 5.9. TMT-Based Proteomics Analysis

Digested peptides were prefractionated with the Ultimate 3000 system (Thermo Fisher Scientific, Waltham, MA, USA). Then, the peptides were combined into 12 fractions and dried by vacuum centrifugation. Finally, peptides were analyzed by online nanospray LC-MS/MS on an Orbitrap Fusion coupled to an EASY-nano-LC system (Thermo Scientific, MA, USA). All LC-MS/MS raw data were identified and analyzed using Proteome Discoverer 2.1 software (Thermo Fisher Scientific, San Jose, CA, USA; version 2.1) and Scaffold Q+ software (version Scaffold4.7.1, Proteome Software Inc., Portland, OR, USA) [72].

### 5.10. GO Function Annotation Analysis

BLAST2GO (version 3.0) was used for the GO function annotation of the ABA response proteins (ARPs) [73].

### 5.11. PPI Network Construction

The PPI network of ARPs was generated using the STRING online database (http://string-db.org (accessed on 20 July 2021)) (version 10.0) [74]. An interaction relation with a combined score >0.4 was considered a significant statistical difference.

### 5.12. Quantification and Statistical Analysis

All statistical data were collected in GraphPad Prism 8.0.2. ANOVA with a two-tailed Student’s *t*-test was used to evaluate statistical differences, with ^ns^ *p* > 0.05, * *p* < 0.05, ** *p* < 0.01, *** *p* < 0.001. All data were reported as mean ± SD [75].

## Data Availability

The data supporting the findings of this study are available in the Supplementary Materials. The mass spectrometry proteomics data were deposited to the ProteomeXchange Consortium via the PRIDE [76] partner repository with the dataset identifier PXD027962.

## Supplementary Materials

The following are available online at https://www.mdpi.com/article/10.3390/ijms221910314/s1.
