# Supplementary material for: Arabidopsis LSH8 Positively Regulates ABA Signaling by Changing the Expression Pattern of ABA-Responsive Proteins
Source: Int J Mol Sci. 2021 Sep 25;22(19):10314. doi: 10.3390/ijms221910314 (PMC8508927; doi:10.3390/ijms221910314)
Supplement: Supplementary file 1 [file ijms-22-10314-s001.zip › supplymentary file.pdf]

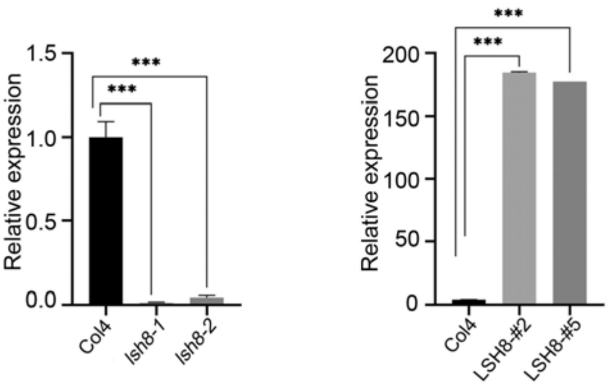

Figure S1. The expression of *LSH8* in Col4, *lsh8* mutants and LSH8 overexpression plants. Imbibed seeds were germinated and grown on 1/2 MS medium for 7 d, and then the seedlings were harvested for QPCR. Data are shown as mean ± SD (n = 3). *ACT2* was used as the internal control.

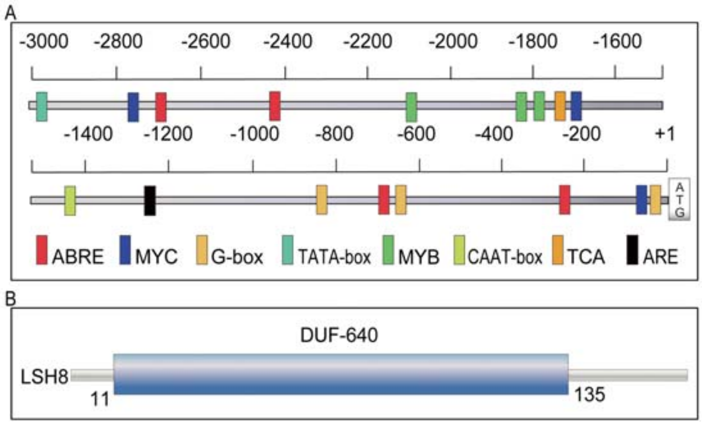

Figure S2. Analysis of *LSH8* promoter and domain. (A) The region of 3,000 bp upstream of the *LSH8* gene was analyzed and shown in colored boxes. The colors in the boxes indicate different cis-acting element ABRE (Red); MYC (Blue); G-box (Buff); TATA-box (Bottle green); MYB (Green) ; CAAT-box (Kelly green); TCA (Orange); ARE (Dark). **(B)** The DUF-640 domain of LSH8 is shown in the blue box, and this domain starts at amino acid 11 of LSH8 and ends at amino acid 135.

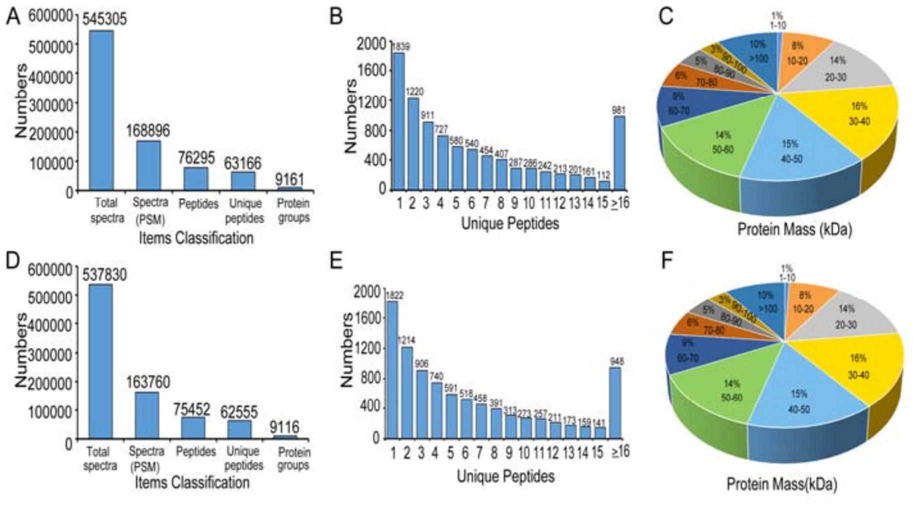

Figure S3. Results of LC-MS/MS identification in the Col4 and *lsh8* mutant. (A, D) Classification of the items used for identifying proteins. **(B, E)** The number of unique peptides that matched to each identified

protein. **(C, F)** Distribution of the average molecular masses of identified proteins.

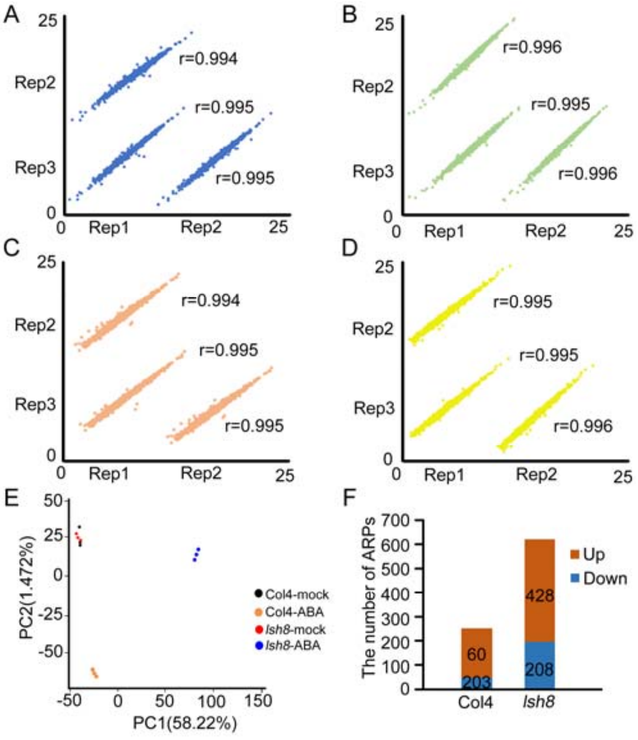

Figure S4. Sample correlation of proteomic data. (A-D) The repeatability of the experiment was verified by data correlation analysis among three replicates. *Pearson* correlation coefficient analysis was used, in which "r" represents the correlation coefficient between replicates of Col4-mock **(A)**, Col4-ABA **(B)**, *lsh8* mutant-mock **(C)** and *lsh8* mutant-ABA **(D)**, respectively. **(E)** Principal component (PCA) analysis. **(F)** The number of ARPs in Col4 and *lsh8* mutant.

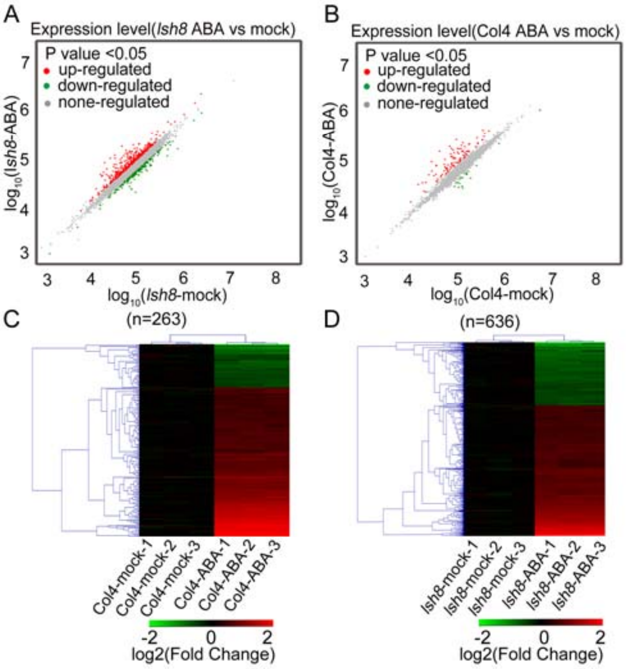

Figure S5. Summary of the proteomic data. (A, B) The overall distribution of protein abundance. The red, green and grey dots represent the up-regulated proteins, down-regulated proteins and none-regulated proteins, respectively. **(C, D)** Heatmap showing the expression of ARPs in Col4 or *lsh8* mutant.

Table S1: Primer used in the study.

Table S2: Basic profiles of proteomics data in wild type Col4.

Table S3: Basic profiles of proteomics data in *lsh8* mutant.

Table S4: Protein change pattern analysis of wild type Col4 under ABA treatment.

Table S5: Protein change pattern analysis of *lsh8* mutant under ABA treatment.
